# High-Precision Optimization of BIM-3D GIS Models for Digital Twins: A Case Study of Santun River Basin

**DOI:** 10.3390/s25154630

**Published:** 2025-07-26

**Authors:** Zhengbing Yang, Mahemujiang Aihemaiti, Beilikezi Abudureheman, Hongfei Tao

**Affiliations:** 1 College of Hydraulic and Civil Engineering, Xinjiang Agricultural University, Urumqi 830052, China; xjnyyzb@outlook.com (Z.Y.); xjaubei@outlook.com (B.A.); taohongfei@xjnydx.wecom.work (H.T.); 2 Xinjiang Key Laboratory of Hydraulic Engineering Security and Water Disasters Prevention, Urumqi 830052, China

**Keywords:** IFC, OSGB, QEM algorithm, LOD

## Abstract

The integration of Building Information Modeling (BIM) and 3D Geographic Information System (3D GIS) models provides high-precision spatial data for digital twin watersheds. To tackle the challenges of large data volumes and rendering latency in integrated models, this study proposes a three-step framework that uses Industry Foundation Classes (IFCs) as the base model and Open Scene Graph Binary (OSGB) as the target model: (1) geometric optimization through an angular weighting (AW)-controlled Quadric Error Metrics (QEM) algorithm; (2) Level of Detail (LOD) hierarchical mapping to establish associations between the IFC and OSGB models, and redesign scene paging logic; (3) coordinate registration by converting the IFC model’s local coordinate system to the global coordinate system and achieving spatial alignment via the seven-parameter method. Applied to the Santun River Basin digital twin project, experiments with 10 water gate models show that the AW-QEM algorithm reduces average loading time by 15% compared to traditional QEM, while maintaining 97% geometric accuracy, demonstrating the method’s efficiency in balancing precision and rendering performance.

## 1. Introduction

In the context of global digital transformation, informatization in water conservancy engineering has positioned digital twin watersheds as a pivotal development direction [1]. Digital twin technology has evolved from theoretical exploration to practical deployment, with models serving as a critical component of digital twin systems and a foundational prerequisite for realizing their applications [2]. Virtual geometric models, created using 3D modeling tools, exhibit high spatiotemporal consistency and visual fidelity with physical entities, forming the foundation of virtual representations [3].

In engineering, Building Information Modeling (BIM) and 3D Geographic Information Systems (3D GIS) have emerged as key modeling techniques [4]. While BIM excels in precise geometric detailing of building components and attribute representation, it faces challenges in large-scale geographic scene processing. Conversely, 3D GIS enables efficient macro-environment rendering but lacks fine-grained representation of building interiors [5,6,7]. The integration of BIM and 3D GIS offers comprehensive 3D data support for digital twin watersheds. Despite these advantages, balancing ‘high-precision modeling’ and ‘efficient rendering’—core requirements for digital twin applications—remains a significant challenge in BIM-3D GIS integration.

Unsimplified BIM models require the loading of massive datasets during each execution, which places significant demands on storage and rendering resources during visualization and spatial analysis—this could potentially lead to system crashes [8,9,10,11]. The technical challenges posed by the fine-grained characteristics of BIM models can be systematically analyzed across two interrelated dimensions. First, at the semantic integration level, the semantic heterogeneity between BIM and 3D GIS must be resolved through multi-faceted approaches: the construction of domain-specific semantic networks [12], the development of custom multi-mesh algorithms [13], the implementation of automated Industry Foundation Classes (IFCs) schema mapping [14], and the enabling of hierarchical data fusion [15]. Second, geometric integration presents two main challenges: (1) Triangular mesh simplification requires algorithms such as vertex decimation [16], edge collapse [17], and adaptive subdivision [18]. While these methods are designed to preserve overall mesh topology, they inherently risk the loss of geometric features in practical applications, thereby compromising model accuracy [19]. (2) BIM-3D GIS integrated models often exceed hundreds of gigabytes or even terabytes, necessitating the use of Level of Detail (LOD) technology to improve rendering performance. However, BIM models lack the mature LOD hierarchy inherent in 3D GIS systems [20], making cross-model dynamic LOD remapping a critical integration challenge. The key to balancing ‘high-precision modeling and efficient rendering’ lies in two complementary strategies: the geometric optimization of BIM components and adaptive LOD hierarchical mapping [21].

In the domain of BIM geometric model simplification, the Quadric Error Metrics (QEM) edge collapse algorithm outperforms vertex decimation methods in preserving the local topology and geometric fidelity of 3D models [22]. Existing research has built upon this foundation through the following modifications: Liang et al. [23] integrated Gaussian curvature with the Whale Optimization Algorithm (WOA-DE); however, the increased iteration count significantly compromises computational efficiency. Ma et al. [24] introduced a weighted QEM algorithm that considers vertex neighborhood area, curvature, and planarity. However, its planarity weighting demonstrates limited adaptability to regions with abrupt curvature changes (e.g., curved gate surfaces in hydraulic structures). Zhang et al. [25] developed a 3D mesh lightweighting algorithm that incorporates curve approximate curvature and the first-order neighborhood triangle area as penalty factors, effectively reducing model deformation while maintaining detailed features—though at the cost of prolonged runtime. Zhang et al. [26] proposed an edge subdivision QEM algorithm that resolves post-collapse facet anomalies through local triangular face reconstruction. Nevertheless, pronounced simplification errors persist in regions with normal vector mutations (e.g., hoist support structures). Notably, current algorithms predominantly use absolute curvature values as the core weighting metric, overlooking the normal vector angle—a direct indicator of geometric ‘sharpness.’ This oversight introduces non-negligible errors in flow field simulations, particularly in scenarios requiring high-precision hydraulic modeling.

In the GIS domain, Open Scene Graph Binary (OSGB) models utilize a mature hierarchical LOD system that dynamically adjusts geometric complexity based on viewing distance, ensuring efficient rendering of large-scale terrain datasets [27]. Conversely, BIM’s LOD framework—defined by the AEC industry’s AIA (American Institute of Architects) standard—prioritizes model completeness across progressive stages (e.g., LOD 400 mandates fabrication-level details), emphasizing information continuity throughout the design and construction phases. This divergence in design philosophy creates integration challenges: while GIS LOD focuses on simplification rates to optimize rendering efficiency, BIM LOD centers on semantic richness for lifecycle management. Existing efforts to bridge this gap have encountered significant limitations. Deng et al. [20] proposed a static mapping scheme between BIM and GIS LOD levels; however, their approach lacks dynamic adaptability to real-time view changes. Varlık [28] achieved semantic and geometric transformation through sequential conversion (IFC to CityGML (City Geography Markup Language) to 3D Tiles using FME), but this multi-step process risks degrading critical geometric features (e.g., curved surfaces of hydraulic gates) and semantic relationships (e.g., component adjacency). Zhu [29] directly processed IFC models using OCCT (Open Cascade Technology) to preserve LOD fidelity; however, manual triangulation parameter tuning is required, making it a labor-intensive approach incompatible with scalable engineering workflows. In flood evolution simulations for water conservancy projects, these limitations become particularly pronounced: static LOD strategies cannot support seamless transitions between basin-wide overviews and localized dyke breach analyses, while intermediary format conversions compromise the high-precision geometric data essential for safety-critical applications.

To address the critical trade-off between high-precision modeling and efficient rendering in BIM-3D GIS integration, this study proposes a three-pronged framework: first, an angle-weighted QEM edge collapse algorithm (AW-QEM) is introduced, incorporating triangular face normal vector angles and vertex curvature as adaptive weighting parameters to maintain geometric fidelity while achieving specified simplification rates, particularly prioritizing edges in high-curvature regions, such as hydraulic component surfaces; Second, a dynamic LOD mapping strategy is established to bridge IFC component visibility attributes with OSGB multi-resolution tiles, leveraging real-time view-dependent scheduling logic to resolve rendering inefficiencies caused by BIM’s static structure; finally, millimeter-level coordinate registration is achieved by combining the seven-parameter transformation method with Scale-Invariant Feature Transform (SIFT) feature matching, ensuring precise alignment from IFC local to OSGB global coordinates. Through this integrated workflow of algorithm optimization, hierarchical mapping, and coordinate registration, the approach enhances data transmission efficiency while preserving sub-centimeter geometric precision, offering a scalable engineering solution for BIM-3D GIS integration, validated in large-scale digital twin projects such as the Santun River Basin.

## 2. Materials

### 2.1. IFC

With the rapid development of BIM technology, various data models and formats have emerged. To ensure the general applicability of our integration approach, this study adopts IFC, an open data standard developed by buildingSMART (https://www.buildingsmart.org) [30]. Based on the EXPRESS language, IFC is a vendor-independent model designed to facilitate seamless BIM data exchange across different software platforms, ensuring information consistency and accuracy.

In BIM, LOD quantifies model completeness; however, LOD-based visualization techniques are not widely adopted in BIM rendering. The AIA has established an LOD standard for the AEC industry, transforming component precision from a vague concept into a structured description. Table 1 summarizes the AIA-defined LOD levels (https://bimforum.org/resource/lod-level-of-development-lod-specification/ (accessed on 1 June 2025)), ranging from conceptual (LOD 100) to as-built (LOD 500) models.

### 2.2. OSGB

OSGB is a binary data format derived from the Open Scene Graph (OSG) open-source rendering engine, designed for the efficient storage and processing of high-precision 3D scene models. Its lightweight processing optimizes data storage, enhances transmission efficiency, reduces costs, and improves rendering performance. OSGB models are typically generated using UAV oblique photography, with post-processing and export conducted through tools such as Context Capture. During export, users define LOD divisions and simplification rates (usually four levels: 0%, 50%, 75%, and 100% simplification by default).

**Figure 1:**
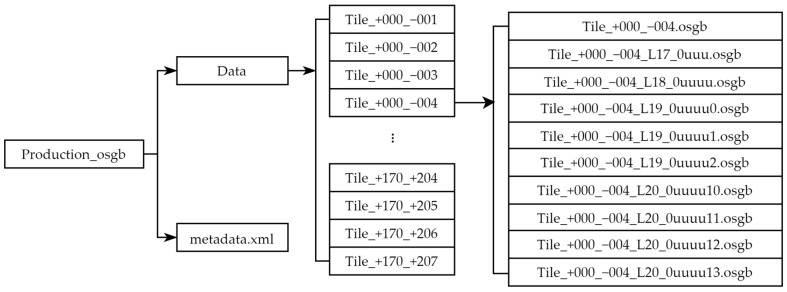
Structure diagram of the OSGB file.

In OSGB files, the data directory serves as the root for model data, containing all tile subdirectories of the scene. Each tile subdirectory, referred to as a root block, stores partial geographic scene data as independent units. Root blocks correspond to different LOD levels in a hierarchical structure—for example, L17 (100% simplification, lowest accuracy), L18 (75%), L19 (50%), and L20 (0%, highest accuracy). This structure enables dynamic loading of model details based on viewing distance during storage and rendering, as illustrated in Figure 1.

### 2.3. BIM-3D GIS Integration Patterns

Three primary approaches exist for BIM-3D GIS integration: embedding BIM into GIS, integrating GIS into BIM, and using third-party systems [31]. The first approach, BIM-to-GIS integration, primarily involves converting IFC to CityGML for GIS visualization. Van et al. [32] unified the Dutch IMGeo 2D standard with CityGML to establish a national 3D geospatial framework, while Kang et al. [33,34] proposed conceptual mappings from IFC to CityGML. However, these methods require simplifying IFC models to fit CityGML’s limited building definitions, resulting in low conversion efficiency and data loss. In contrast, GIS-to-BIM integration is rarely employed due to challenges in translating GIS semantics and geographic geometry into BIM-compatible formats. Studies on IFC-CityGML conversion primarily focus on geometric extraction and semantic transformation, but irreversible information loss during conversion impedes model interoperability. Given these limitations, third-party system integration has emerged as a viable alternative. Demir et al. [35] achieved loose-coupling integration for automated zoning compliance checking, while Zhang et al. [36] developed the BGIP platform to unify BIM and 3D GIS for hydropower construction applications.

## 3. Model Integration Methodology

### 3.1. Integration Architecture

**Figure 2:**
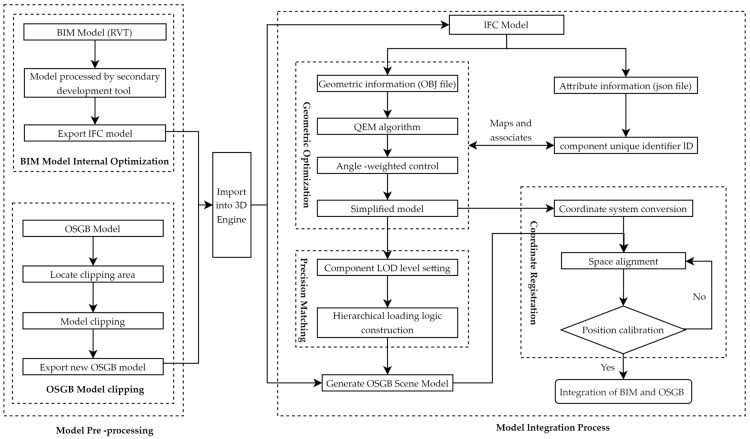
Integration framework of IFC model and OSGB model.

This study integrates BIM and 3D GIS models using third-party 3D engines (e.g., Unity, UE), with the integration framework illustrated in Figure 2. The process consists of four key steps: (1) Preprocessing of IFC and OSGB models: For BIM (RVT) models, secondary development tools are used to process, export, and optimize IFC models. Concurrently, the OSGB model undergoes spatial querying to locate the clipping region based on the geographic coordinates of the BIM model, followed by cropping and exporting a new OSGB model with the redundant terrain tiles removed. (2) Model import and geometric optimization: The preprocessed models are imported into the 3D engine. The geometric information of the IFC model (converted to OBJ files) is simplified using the QEM algorithm with angular weighting control, while attribute information (stored as JSON files) is linked to the geometric data through unique component identifiers (IDs). (3) LOD hierarchical mapping: Based on the OSGB model’s LOD definition, a LOD classification scheme is configured for the IFC model components. A hierarchical loading logic is established to generate a new scene model, enabling dynamic switching of model details based on the viewing distance. (4) Coordinate system registration: The local coordinate system of the IFC model is transformed into the global coordinate system of the OSGB model. Spatial alignment is achieved through precise coordinate transformation and feature matching, finalizing the integration of the IFC and OSGB models.

### 3.2. Model Preprocessing

During BIM project model development, numerous unused elements are generated. Before exporting the IFC model, secondary development tools in BIM software (Revit 2024) are used to remove redundant data, thereby enhancing model data quality. The OSGB model includes texture maps for the entire geographic scene, which may cause building outline overlaps when integrated with structural BIM models. To address this, OSGBLab (Hebei, China) performs spatial queries based on the BIM model’s geographic coordinate range, removes the corresponding area tiles from the OSGB model, and retains the remaining data to generate a new OSGB file.

### 3.3. Geometric Model Optimization

This section describes the edge collapse algorithm [37] and the QEM algorithm [38], and then presents an approach to adjusting the edge collapse order using angular weighting. This method aims to enhance geometric feature preservation during mesh simplification by dynamically prioritizing edge collapses that retain critical angular and curvature characteristics.

#### 3.3.1. Edge Collapse Algorithm

**Figure 3:**
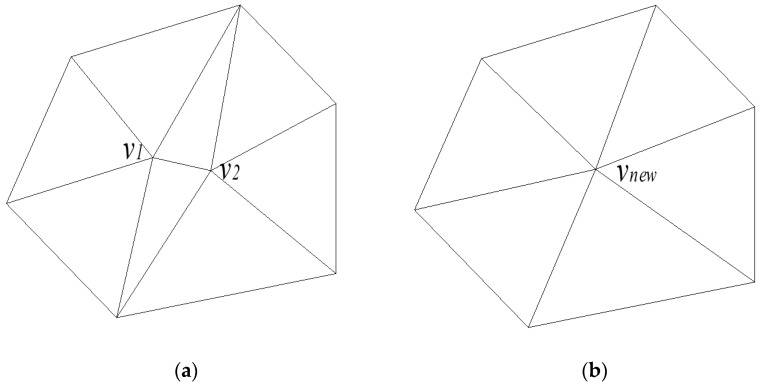
Schematic diagram of the edge collapse algorithm, where (**a**) represents the schematic diagram of the original model’s mesh and (**b**) represents the schematic diagram after edge collapse.

The edge collapse algorithm consists of three main steps: calculating the collapse cost of each edge in the triangular mesh, sorting these edges based on cost metrics, and preferentially collapsing edges with lower costs. As illustrated in Figure 3, when the edge between vertices *v*_1_ and *v*_2_ is collapsed, a new vertex, *v_new_*, is generated. Subsequently, all points originally connected to *v*_1_ and *v*_2_ are reconnected to *v_new_*. Finally, degenerate triangles are removed to preserve the integrity of the triangular mesh model.

#### 3.3.2. QEM Algorithm

The QEM algorithm uses the squared Euclidean distance from a vertex to its incident planes as the error metric. Specifically, for any new vertex *v_new_* in the mesh, the error value Δ(*v*) is defined as the sum of squared distances from *v_new_* to each plane of the triangular faces in the set Planes(*v*)—the collection of planes incident to vertex *v*—as formulated in Equation (1):

(1) $$\Delta (v)=\sum_{p\in \mathrm{planes}(v)}d_{p}^{2}(v)=\sum_{p\in \mathrm{planes}(v)}{\left(p^{T}v\right)}^{2}=\sum_{p\in \mathrm{planes}(v)}v^{T}\left(pp^{T}\right)v=v^{T}\left(\sum_{p\in \mathrm{planes}(v)}K_{p}\right)v$$

where $v={\left(v_{x}\;v_{y}\;v_{z}\;1\right)}^{T}$ and $p={\left(a\;b\;c\;d\right)}^{T}$ represent the plane of each triangle facet in Planes(*v*), with the plane equation given by ax+by+cz+d=0 and the condition $a^{2}+b^{2}+c^{2}=1$; *Kp* is the quadratic error matrix of plane P, as formulated in Equation (2):

(2) $$K_{p}=pp^{T}=\left(\begin{array}{llll} a^{2} & ba & ca & da\\ ab & b^{2} & cb & db\\ ac & bc & c^{2} & dc\\ ad & bd & cc & d^{2} \end{array}\right)\;$$

Define the quadratic error matrix of vertex *v* as $Q_{v}=\sum_{p\in \mathrm{planes}(v)}K_{p}$. Taking Figure 3 as an example, when vertices *v*_1_ and *v*_2_ collapse into *v_new_*, their collapse cost is expressed as formulated in Equation (3):

(3) $$\Delta (v)=v^{T}\left(\sum_{p\in \mathrm{planes}(v1)}K_{p}+\sum_{p\in \mathrm{planes}(v2)}K_{p}\right)v=v^{T}(Q_{v1}+Q_{v2})v$$

#### 3.3.3. Angle-Weighting

**Figure 4:**
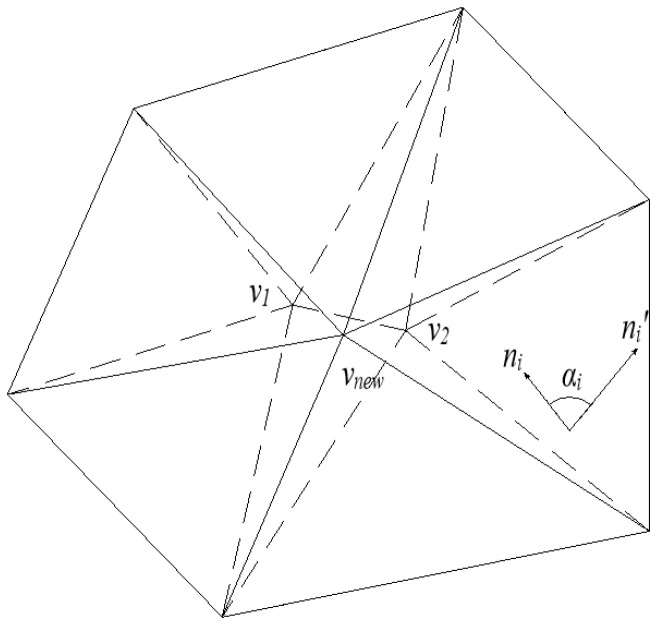
Schematic diagram of the rotation of triangular facets.

Although the QEM algorithm can preserve the general shape of the model to some extent, it often diminishes or even loses the sharp geometric features of components, reducing geometric accuracy. The angular rotation of triangular facets significantly impacts the sharpness of geometric features—excessive rotation angles not only reduce sharp edges but can also cause facet flipping. This results in undesired transitions, such as gaps and overlaps between adjacent facets, compromising geometric precision, as illustrated in the angular rotation diagram of triangular facets in Figure 4. To address this, the study incorporates the facet rotation angle before and after edge collapse as a weight parameter in the QEM algorithm, hereinafter referred to as the Angle-Weighting QEM (AW-QEM) algorithm. By adjusting the edge collapse order, this approach preserves component edge features and mitigates the deterioration of geometric features.

For any triangular facet with vertices *v*_1_, *v*_2_, and *v*_3_, the normal vector calculation formula is given by Equation (4). After edge collapse, the maximum angle of normal vector change for triangular facets in the neighborhood is denoted as *α*_max_, with its expression provided by Equation (5):

(4) $$n_{i}=\frac{\left(V_{2}-V_{1}\right)\times \left(V_{3}-V_{2}\right)}{\left\|\left(V_{2}-V_{1}\right)\times \left(V_{3}-V_{2}\right)\right\|}$$

(5) $$\alpha_{\max}=\max\left\{\begin{array}{c} \arccos\left(n_{0}\times n_{0}^{\prime }\right)\\ \arccos\left(n_{1}\times n_{1}^{\prime }\right)\\ \vdots \\ \arccos\left(n_{i}\times n_{i}^{\prime }\right) \end{array}\right\}$$

In the equations, *n_i_* represents the unit normal vector of triangular faces in the neighborhood before edge collapse, and *n_i_′* denotes the unit normal vector of triangular faces in the neighborhood after edge collapse. In 3D models, vertex curvature reflects the shape characteristics of the local region surrounding the vertex. The approximate vertex curvature is used as a control parameter, defined as shown in Equation (6). Edges containing vertices with high curvature are assigned to lower collapse priorities, while edges with low curvature are prioritized for collapse. This ensures that model simplification begins in relatively unimportant regions and progresses to critical areas, preserving the model’s sharp features.

(6) $$C_{V_{i}}=\frac{\sum_{k}\alpha \left(n_{V_{i}},n_{i}\right)}{k}$$

In the equation, *n_vi_* is calculated through area-weighted normalization of the normal vectors of triangular faces associated with the vertex, where the weight is the area *S_i_* of each face, as shown in Equation (7):

(7) $$n_{V_{i}}=\frac{\sum_{i=1}^{k}S_{i}n_{i}}{\left\|\sum_{i=1}^{k}S_{i}n_{i}\right\|}$$

Here, $\alpha \left(n_{V_{i}},n_{i}\right)$ represents the angle between the vertex normal vector *n_vi_* and the *k*-th associated triangular face.

Taking the ratio of the approximate vertex curvature *C_Vi_* to *α*_max_ as the weight parameter, we substitute it into Equation (1) and use the result as the final collapse cost for edge collapse in the AW-QEM algorithm, as shown in Equation (8):

(8) $$\Delta (v)=V^{T}\left(\frac{C_{V}\sum_{p\in \mathrm{planes}(v)}K_{p}}{\alpha_{\max\;}}\right)V=V^{T}QV$$

### 3.4. LOD Hierarchical Mapping

The OSGB model architecture uses multi-resolution LOD technology to construct a spatial data pyramid, with the core feature being differentiated detail representation through grid-based partitioning. Specifically, this technology divides large-scale terrain and scenes into regular grid cells using spatial indexing, assigning each cell a resolution level based on its visual importance. The pyramid employs a dynamic scheduling strategy during rendering: low-resolution levels are automatically activated when the camera is distant to reduce computational load, while high-resolution levels are engaged for close views to preserve geometric detail, thereby optimally balancing visual fidelity and rendering efficiency [39].

**Figure 5:**
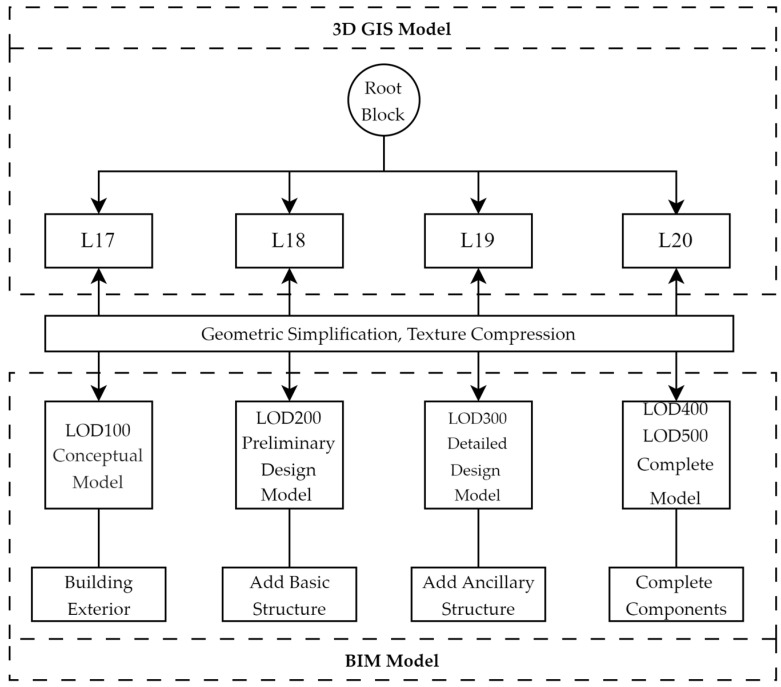
LOD matching scheme for IFC and OSGB models.

Using the OSGB root node as a reference and following its 4-level LOD system (L17–L20), the hierarchical LOD matching scheme in Figure 5 configures component visualization effects. Geometric simplification algorithms then perform multi-precision simplification on IFC building components, with differentiated simplification ratios defined according to component type:

- L17 (large-area watershed display): 100% simplification (no simplification applied to IFC models);
- L18: 50% simplification to retain critical geometric features;
- L19: 25% simplification for visually accessible components, 50% for non-visible components;
- L20 (highest precision): preserves the original model accuracy.

IFC models with varying simplification ratios are mapped to the OSGB scene root block, redefining the dynamic scheduling logic for each LOD level. Both IFC and OSGB models are classified based on predefined viewing distances, enabling real-time grid switching during rendering according to the observer’s viewpoint.

### 3.5. Coordinate Registration

#### 3.5.1. Coordinate System Transformation

Components in the IFC model use their geometric center or characteristic point as the coordinate origin, establishing an independent local coordinate system. In contrast, the OSGB model utilizes a global geodetic coordinate system to enable seamless integration of scene-level 3D data under a unified geospatial benchmark. The fundamental discrepancy between these two coordinate systems results in issues such as misaligned superposition of building models with real-world terrain, failed LOD spatial indexing, and errors in analytical calculations using the integrated model. The conversion of the IFC model’s local coordinates to the global system is achieved through the transformation matrix *T_t_* (Equations (9)–(13)).

(9) $$T_{t}=R_{x}R_{y}R_{z}T$$

(10) $$R_{x}=\left[\begin{array}{cccc} 1 & 0 & 0 & 0\\ 0 & \cos\theta_{x} & \sin\theta_{x} & 0\\ 0 & -\sin\theta_{x} & \cos\theta_{x} & 0\\ 0 & 0 & 0 & 1 \end{array}\right]$$

(11) $$R_{y}=\left[\begin{array}{cccc} \cos\theta_{y} & 0 & -\sin\theta_{y} & 0\\ 0 & 1 & 0 & 0\\ \sin\theta_{y} & 0 & \cos\theta_{y} & 0\\ 0 & 0 & 0 & 1 \end{array}\right]$$

(12) $$R_{z}=\left[\begin{array}{cccc} 1 & 0 & 0 & 0\\ 0 & \cos\theta_{z} & \sin\theta_{z} & 0\\ 0 & -\sin\theta_{z} & \cos\theta_{z} & 0\\ 0 & 0 & 0 & 1 \end{array}\right]$$

(13) $$T=\left[\begin{array}{cccc} 1 & 0 & 0 & 0\\ 0 & 1 & 0 & 0\\ 0 & 0 & 1 & 0\\ x_{0} & y_{0} & z_{0} & 1 \end{array}\right]$$

where (*x*_0_, *y*_0_, *z*_0_) represents the origin of the local coordinate system in the IFC model, *R_x_*, *R_y_*, and *R_z_* denote the rotation matrices about the X, Y, and Z axes, respectively, and *θ_x_*, *θ_y_*, and *θ_z_* signify the angles between the rotation vector and the X, Y, and Z axes, respectively.

#### 3.5.2. Spatial Alignment

During spatial positioning, while BIM models can be placed in corresponding positions within the OSGB model through direct translation and rotation, this method relies on the operator’s experience and visual judgment, making it difficult to achieve centimeter- or millimeter-level precision alignment. Using the OSGB model’s coordinate system as the reference, transformation parameters are accurately calculated based on homologous feature points. These points have distinct geometric characteristics and easily determinable coordinates (e.g., building corner points). Subsequently, the local coordinate system of the BIM model is precisely transformed into the reference system using these parameters. Finally, spatial position calibration is performed through feature matching to achieve high-precision spatial alignment. The specific steps are as follows:

(1) Homologous feature points: At least three prominent and easily identifiable homologous feature points—geometrically distinct points with unambiguous coordinates (e.g., building vertices or structural intersections)—are selected in both BIM and OSGB models. In the OSGB model, the coordinates of these common points are denoted as (*X_Oi_*,*Y_Oi_*,*Z_Oi_*), with corresponding coordinates in the BIM model as (*X_Bi_*,*Y_Bi_*,*Z_Bi_*), where *i* = 1, 2,···, *n* and *n* represents the number of common points.

(2) Spatial alignment: Based on these common points, coordinate transformation equations (Equation (14)) are established using the seven-parameter Helmert transformation model. The least squares method is applied to minimize the sum of squared residuals between corresponding points, solving for the seven unknown parameters: three translation vectors, three rotation angles, and one scale factor. Once these parameters are determined, they are used to convert the coordinates of all points in the BIM model, enabling precise positioning and alignment within the OSGB model’s geospatial framework.

(14) $$\left[\begin{array}{c} X_{O}\\ Y_{O}\\ Z_{O} \end{array}\right]=(1+m)\cdot R\left(\omega_{x},\omega_{y},\omega_{z}\right)\cdot \left[\begin{array}{c} X_{B}\\ Y_{B}\\ Z_{B} \end{array}\right]+\left[\begin{array}{l} \Delta X\\ \Delta Y\\ \Delta Z \end{array}\right]$$

where *m* is the scale parameter; *ω_x_*, *ω_y_*, and *ω_z_* denote the rotation angles about the *X*, *Y*, and *Z* axes, respectively; and ∆*X*, ∆*Y*, and ∆*Z* represent the translation offsets along the *X*, *Y*, and *Z* axes, respectively.

(3) Position calibration: The SIFT algorithm is applied to extract feature descriptors from key points in both models for cross-model matching. Specifically, the Euclidean distance measures the similarity score between descriptors, and the matching criterion follows Lowe’s ratio test: the ratio of the nearest-neighbor distance to the second-nearest-neighbor distance is used to filter ambiguous matches. The core function of the algorithm is defined as shown in Equations (15) and (16):

(15) $$dist(A,B)=\sqrt{\overset{n}{\underset{i=1}{\sum }}{\left(a_{i}-b_{i}\right)}^{2}}$$

(16) $$\frac{dist\left(A,B_{1}\right)}{dist\left(A,B_{2}\right)}<T$$

where *A* represents a feature descriptor from the BIM model; *B*_1_ and *B*_2_ denote the nearest and second-nearest neighbor descriptors in the OSGB model’s feature descriptor set, respectively; *dist* is the Euclidean distance between descriptors; *n* is the descriptor dimension; *a_i_* and *b_i_* are the *i*-th elements of descriptors *A* and *B*, respectively; and *T* is the predefined matching threshold.

## 4. Case Validation

**Figure 6:**
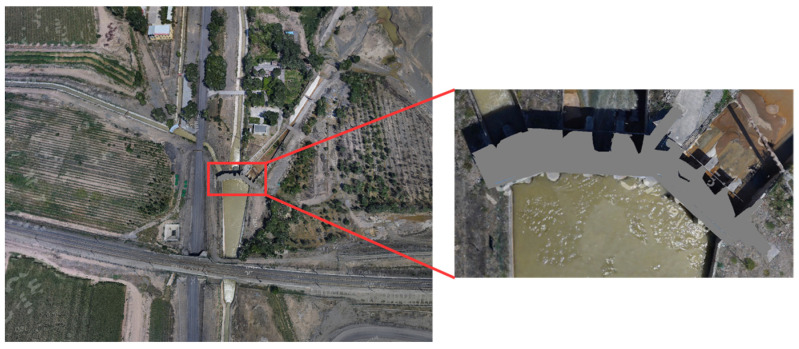
Scene model of a water diversion sluice in Santun River Basin.

The proposed model integration framework was implemented in the Santun River Basin, Xinjiang. The OSGB model, spanning 140 GB and comprising 6279 root block files, represents a 50-km-long basin scene. Focusing on the water diversion gate structure, its contour was extracted as the target area, which was then clipped and geometrically flattened. As illustrated in Figure 6, the rectangular region highlights the precise location of the water diversion gate within the basin model.

**Figure 7:**
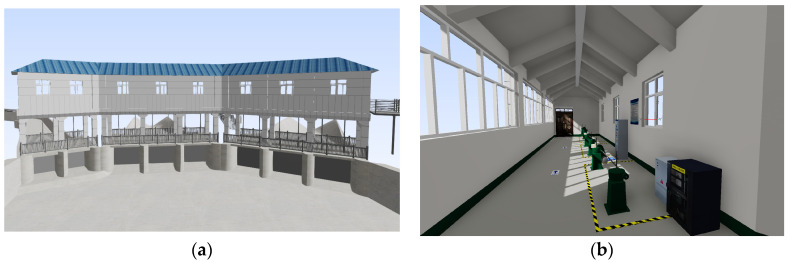
The BIM model of a water diversion sluice, with (**a**) depicting the building exterior and (**b**) illustrating the indoor layout.

In this project, ten BIM models of water gate control stations were developed using Revit^®^ 2024 software based on the Santun River Basin engineering drawings, with model files totaling 776 MB in size. Model texture mapping data were acquired through on-site photography and professionally processed in Adobe Photoshop^®^ as texturing materials, ensuring accurate visual fidelity when integrated with the OSGB scene. Taking one control station as an example, its indoor/outdoor equipment and texture materials in the BIM model closely replicate real-world conditions, as shown in the detailed view in Figure 7.

Unity was selected as the 3D engine platform, with C# programming used to implement OSGB model import, IFC data-model decoupling, IFC geometric model optimization, precision synchronization, and coordinate unification. The experiment utilized a standard laptop configuration: Intel^®^ Core™ i5-7300HQ 2.50GHz CPU, 16.0 GB RAM, and a 1 TB hard disk (Santa Clara, CA, USA), with development environments set to Unity Editor 2022.3 and Visual Studio 2022. The entire integration process took approximately 1 h, with step-wise timings as follows:

- OSGB model import: ~5 min
- Import and optimization of ten control station BIM models: ~30 min
- LOD accuracy matching: ~10 min
- Model spatial coordinate calculation after manual homologous feature point selection: ~15 min

These timings were considered optimal for the described workflow.

To ensure reliability and consistency, each test—including model loading time, LOD visualization, coordinate accuracy, and geometric optimization algorithm comparisons—was repeated five times across six similar hardware configurations under identical software settings. The reported values represent the averages of thirty runs, with variances ranging from 0.01 to 0.03, demonstrating the proposed method’s excellent stability and reproducibility.

### 4.1. LOD Visualization

**Figure 8:**
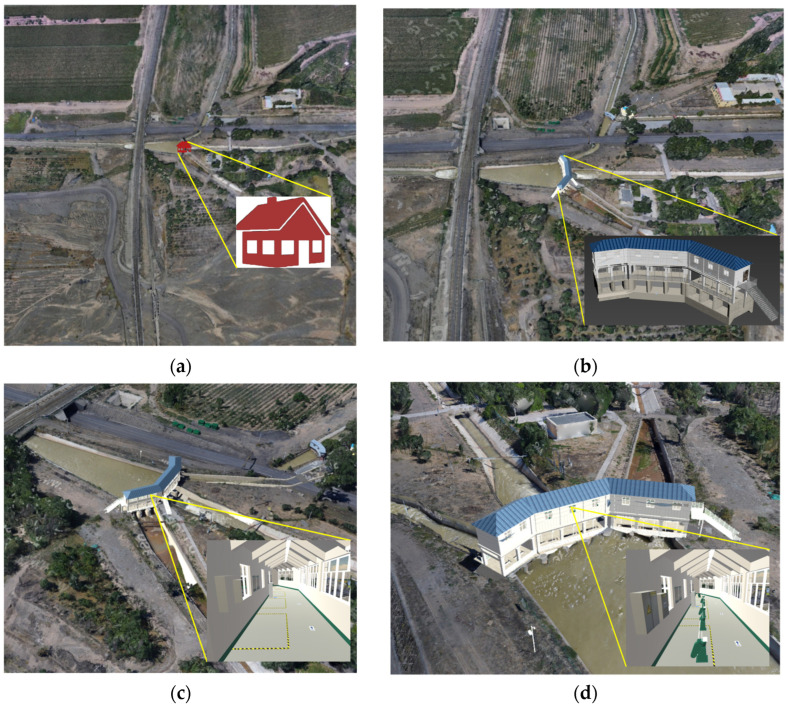
Hierarchical LOD representation of the integrated model. (**a**) L17 (Symbolic Level): Simplified symbolic representation focusing on overall structure; (**b**) L18 (Basic Outdoor Level): Key outdoor components (roof, walls, stairs) with 50% geometric simplification; (**c**) L19 (Enhanced Indoor–Outdoor Level): Progressive loading of indoor equipment with balanced simplification (25% outdoor, 50% indoor); (**d**) L20 (Full Detail Level): Full-component display with uniform 25% simplification to maintain geometric fidelity.

Taking a water diversion sluice as an example, the AW-QEM algorithm was applied to its BIM geometric model, achieving a final simplification ratio of 25%. The LOD hierarchical representation of the integrated model is shown in Figure 8:

- L17 (Symbolic Level): The water diversion sluice model is represented in a symbolic form, omitting detailed component geometry to prioritize overall structural visibility.
- L18 (Basic Outdoor Level): Main outdoor components—including the roof, outer walls, floors, and stairs—are loaded, with their geometric shapes simplified by 50% to retain essential structural outlines while reducing computational load.
- L19 (Enhanced Indoor–Outdoor Level): As the LOD scale increases, key indoor equipment components (e.g., hoists, control cabinets, fire hydrants) are progressively loaded. Outdoor components undergo a stricter simplification ratio of 25%, while indoor components maintain a 50% simplification ratio to balance detail retention and performance.
- L20 (Full Detail Level): All scene components are fully loaded, with a uniform 25% simplification ratio applied to preserve geometric fidelity across both indoor and outdoor elements.

### 4.2. Coordinate Accuracy

**Figure 9:**
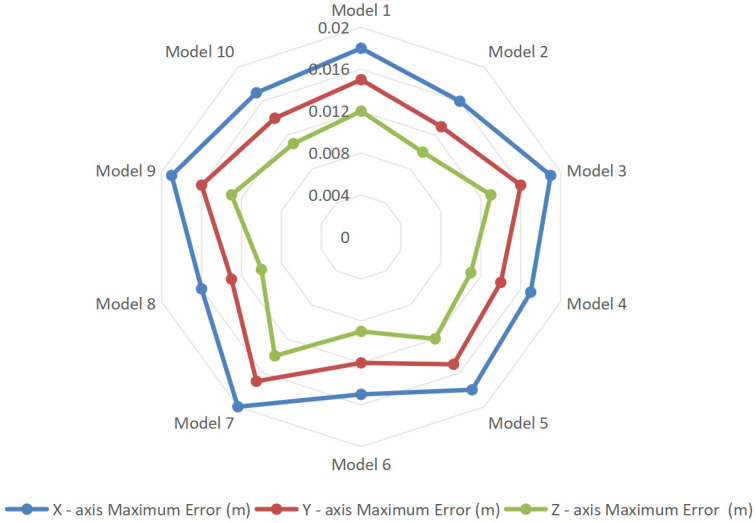
Coordinate accuracy errors.

Coordinate accuracy verification was conducted on the corner points of each water diversion sluice building and the midpoints of each building edge. The coordinate accuracy errors of the model are presented in Figure 9. The verification results show that the errors along the X, Y, and Z axes all remain below 0.02 m. Specifically, the maximum error in the X-axis position is 0.02 m, the maximum error on the Y-axis is 0.017 m, and the maximum error on the Z-axis is 0.014 m. These findings confirm that the proposed method demonstrates high accuracy in practical applications.

### 4.3. Comparison of Geometric Model Optimization Algorithms

For the ten water diversion sluice models in this study, a uniform LOD precision synchronization scheme was applied, and comparative experiments were conducted using the QEM algorithm, the vertex clustering algorithm, and the proposed AW-QEM algorithm.

**Figure 10:**
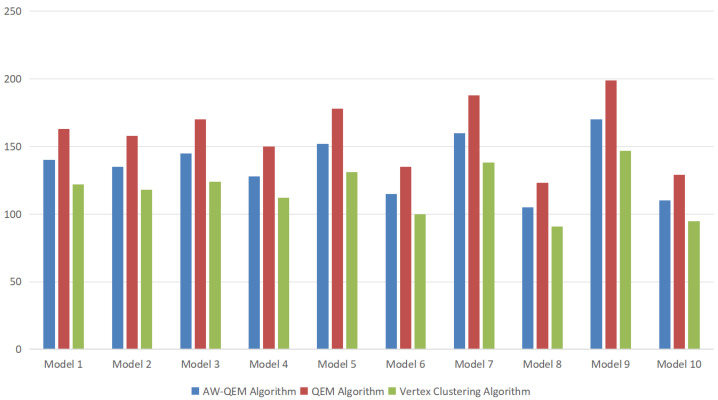
Model loading time comparison.

**Figure 11:**
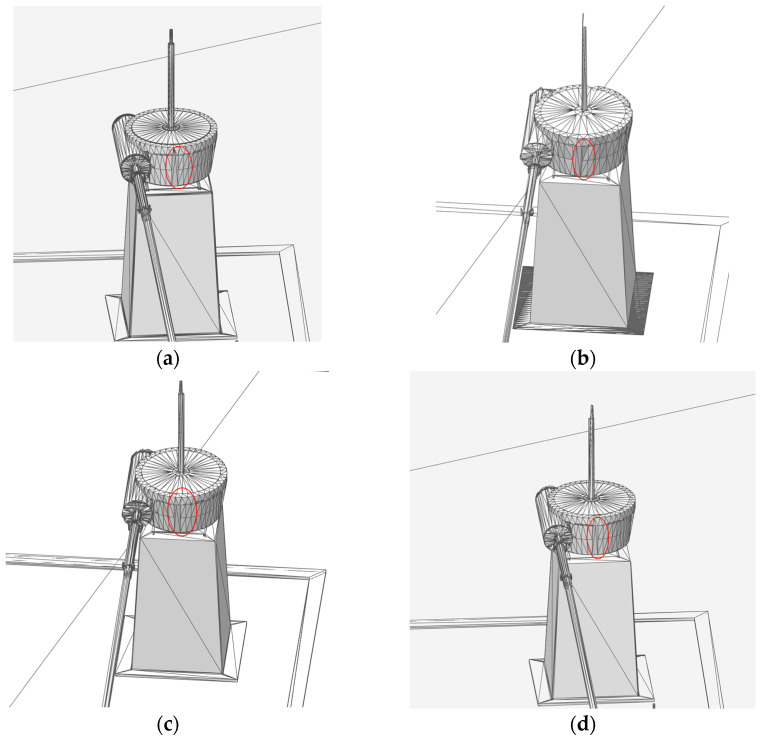
The mesh optimization comparison of three algorithms for a hoist, with (**a**) depicting the initial model, (**b**) the result of the vertex clustering algorithm, (**c**) the outcome of the QEM algorithm, and (**d**) the effect of the proposed AW-QEM algorithm. These images illustrate the visual differences in mesh simplification and feature preservation among the methods. Red circles indicate the key changed areas.

Model loading times are compared in Figure 10, while mesh optimization differences for a hoist component are visualized in Figure 11. Table 2 summarizes the average simplification ratio, geometric accuracy, and loading time derived from the experimental data.

Experimental results indicate that although the vertex clustering algorithm achieves a higher simplification ratio, it leads to significant geometric feature loss for pump-sluice equipment (see Figure 11b), with moderate accuracy performance. This makes it suitable only for low-precision scenarios. The AW-QEM and QEM algorithms exhibit comparable simplification ratios (Figure 11c,d), but the proposed AW-QEM algorithm outperforms both in geometric feature preservation and loading efficiency. It maintains component-level visual fidelity while accelerating model loading, making it better suited for high-precision, high-realism digital twin applications.

Quantitative analysis shows that by integrating vertex curvature and maximum rotation angle weighting, the AW-QEM algorithm effectively retains 3D model detail features while reducing the triangular mesh count. Notably, it mitigates the impact of geometric complexity on loading performance, achieving a 15% reduction in loading time compared to the traditional QEM algorithm.

## 5. Discussion

Compared with traditional BIM-GIS integration methods (such as IFC-to-CityGML conversion [32,33,34]), this study avoids information loss and inefficiency caused by format conversion, achieving heterogeneous model fusion through third-party engines, which is better suited for complex scenarios in water conservancy basins.

The core innovation lies in introducing angle weighting into the QEM edge collapse algorithm, preserving geometric features by dynamically adjusting the collapse order. As shown in Table 3, we compare the AW-QEM algorithm with the WOA-DE algorithm proposed by Liang [23] and the edge subdivision QEM algorithm proposed by Zhang [26]. The AW-QEM algorithm directly measures feature sharpness through *α*_max_ (the maximum normal vector angle of adjacent triangular faces). When *α*_max_ exceeds the threshold, the collapse priority of corresponding edges is forcibly reduced, preventing the smoothing of sharp features such as gate edges and hoist supports (as shown in Figure 11c,d). By using the ratio of *α*_max_ to *C_Vi_* (vertex curvature) as the weight, ‘dual-parameter collaborative control’ is achieved—edges with high curvature and high *α*_max_ (such as building corners) are minimally collapsed, while edges with low curvature and low *α*_max_ (such as planar walls) are simplified preferentially. This advantage is particularly significant in visualizing complex components in water conservancy projects (such as hoists). Traditional algorithms often result in the loss of sharp features (Figure 11b), whereas AW-QEM effectively preserves component edges and details (Figure 11d), meeting the high-precision model requirements of digital twins.

In terms of dynamic LOD scheduling, existing GIS LOD systems are designed for single models, whereas this study establishes a bidirectional mapping mechanism between IFC component visibility and the OSGB hierarchy to achieve dynamic scheduling based on different viewing distances: low-precision simplified models are used for distant scenes (L17–L18) to reduce computational load, while high-precision details are loaded for close-range views (L19–L20), balancing rendering efficiency and visual fidelity. Compared with the static simplification strategy of traditional GIS LOD, the bidirectional LOD mapping mechanism proposed herein better meets the ‘high-fidelity for key equipment’ requirement in water conservancy projects.

In terms of coordinate registration, a coordinate transformation matrix (Equations (9)–(13)) is constructed to convert IFC local coordinates to global benchmarks, innovatively integrating the seven-parameter transformation method with the SIFT feature matching algorithm to form a ‘parameter solving-feature fine-tuning’ alignment process. In the Santun River Basin case study in Xinjiang, a combination of manually deployed control points and SIFT automatic matching was applied to texture-deficient areas, such as concrete dams, controlling X/Y/Z axis coordinate errors within 0.02 m (Figure 9). This registration method provides a spatial reference for flood evolution simulations in digital twin watersheds, meeting the high-precision requirements for water conservancy safety analysis.

The BIM-3D GIS integration framework proposed in this study has demonstrated its effectiveness with 10 water gate models from the Santun River Basin; however, the structural diversity of cases and cross-domain applicability still offer room for expansion. In terms of method generality, the geometric optimization strategy based on the angle-weight QEM algorithm (such as the dual-parameter control of *α*_max_ and curvature *C_Vi_*) can be applied to complex structures like bridge trusses and high-rise building curtain walls. For instance, sharp features of bridge steel box girder joints can be protected from collapse smoothing via the *α*_max_ threshold, while curved arch ribs can rely on *C_Vi_* for dynamic simplification priority adjustment. The dynamic LOD matching mechanism also shows potential in viaduct modeling for transportation, enabling hierarchical rendering from urban-level macro-planning (L17–L18) to bearing bolt details (L20) through four-level simplification rates.

In cross-domain expansion, the coordinate registration scheme (seven-parameter method + SIFT feature matching) can be directly applied to city-level BIM-OSGB integration, achieving millimeter-level alignment by capturing building corners and municipal control points. In smart transportation scenarios, the fusion of road BIM models and tilt photography data can utilize the AW-QEM algorithm to optimize components like guardrails and signboards, with LOD levels dynamically scheduled based on viewing distance. However, the existing framework has not addressed the multi-domain semantic gap issue (such as attribute mapping between IFC and transportation-oriented CityGML), nor does it support LOD for dynamic scenarios (e.g., traffic flow, utility tunnel water-level changes)—important future research directions that will integrate ontology and real-time data stream processing technologies.

## 6. Conclusions

To address the challenge of achieving ‘high-precision modeling and efficient rendering’ in BIM-3D GIS integration for digital twin river basins, this study presents a framework for integrating building BIM models with 3D GIS (OSGB) models. The framework is validated through a case study in Xinjiang’s Santun River Basin, utilizing geometric model optimization, LOD hierarchical mapping, and coordinate registration. The key findings are as follows:

(1) For BIM model geometric optimization, an angle-weighted extension of the QEM algorithm (AW-QEM) is introduced to dynamically control the triangular mesh collapse order. This method reduces the model’s data volume while preserving component-level visual fidelity, ensuring that critical geometric features (e.g., sharp edges of water conservancy equipment) remain intact during simplification.
(2) A hierarchical mapping mechanism is established between IFC component visibility and OSGB multi-resolution levels, defining a four-level (L17–L20) dynamic scheduling strategy for simplification ratios. This approach balances macroscopic basin-scale display (low-resolution L17–L18 for reduced computational load) with detailed component-level rendering (high-resolution L19–L20 for feature retention), enabling adaptive visualization across varying viewing distances.
(3) The local coordinate system of IFC models is transformed into the OSGB global geodetic system using a seven-parameter Helmert transformation combined with SIFT feature matching. This integration achieves millimeter-level spatial alignment, resolving coordinate benchmark discrepancies and providing a unified geospatial framework for cross-model analysis, thereby enabling the seamless fusion of BIM components with large-scale 3D GIS terrain.

## Figures and Tables

**Table 1:** The AIA-defined LOD levels ranging from conceptual (LOD 100) to as-built (LOD 500) models.

LOD Level	Name	Definition
LOD 100	Conceptual Model	Uses basic cuboid components as placeholders to represent only the approximate location, shape, and dimensions of objects, without including specific building components or detailed information.
LOD 200	Preliminary Design Model	Builds on LOD 100 by adding basic attribute information (e.g., material properties) and assigning basic texture elements to model components.
LOD 300	Detailed Design Model	Further refines LOD 200 by incorporating detailed geometric shapes, dimensions, and construction attribute information for model elements, with more precise connectivity relationships between elements.
LOD 400	Component Model	Includes all information from LOD 300 and achieves the highest geometric precision, containing manufacturing details, material specifications, and assembly information for model elements.
LOD 500	As-Built Model	Based on LOD 400, incorporate change orders and detailed information required for the operation and maintenance phase. This level represents the highest precision of the data model without altering the geometric representation.

**Table 2:** Comparison of geometric model optimization algorithms.

Comparison Index	AW-QEM	QEM	Vertex Clustering
Loading Time (s)	140 s	165 s	120 s
Optimization Rate (%)	25%	26.54%	35%
Accuracy (%)	97%	93%	80%

**Table 3:** Comparison of principles and functions of three algorithms.

Comparison Dimension	WOA-DE Algorithm	Edge Subdivision QEM Algorithm	AW-QEM Algorithm
Core Principle	Gaussian curvature + QEM	Edge subdivision + quadric error metrics	Angle weighting + vertex curvature
Algorithm Function	Solving via Whale Optimization Algorithm	Triangular facet anomaly repair	Dynamic adjustment of edge collapse order
Feature Preservation Mechanism	Global curvature homogenization (focus on mesh quality)	Local edge subdivision to avoid triangular facet flipping	Weighted by *α*_max_ to *C_Vi_* to prioritize the preservation of sharp edges
Computational Complexity	O(*n*^2^) (complex)	O(*n* log *n*) (simple)	O(*n* log *n*) (simple)

## Data Availability

The original contributions presented in this study are included in the article. Further inquiries can be directed to the corresponding author.

## Abbreviations

The following abbreviations are used in this manuscript:

3D GIS	3D Geographic Information System
IFCs	Industry Foundation Classes
OSGB	Open Scene Graph Binary
QEM	Quadric Error Metrics
AW-QEM	Angle-Weighting Quadric Error Metrics
LOD	Level of Detail
RVT	Revit Model File
OBJ	Object File
JSON	JavaScript Object Notation
Unity	Unity 3D Engine
CityGML	City Geographic Markup Language
SIFT	Scale-Invariant Feature Transform
AEC	Architecture, Engineering, and Construction
AIA	American Institute of Architects
OSG	Open Scene Graph
AOI	Area of Interest
